# Celiac trunk angiography with balloon occlusion of splenic artery for diagnosis and treatment of splenic steal syndrome

**DOI:** 10.1016/j.radcr.2024.11.052

**Published:** 2024-12-15

**Authors:** Jocelyn Wu, Justin Kim, Kwabena Yamoah, Lealem Aderie, Nariman Nezami, Adam Fang

**Affiliations:** a University of Maryland School of Medicine, Baltimore, MD, USA; b University of Maryland School of Medicine, Department of Diagnostic Radiology and Nuclear Medicine, Division of Vascular and Interventional Radiology, Baltimore, MD, USA; c MedStar Georgetown University Hospital, Washington, DC, USA

**Keywords:** Splenic steal syndrome, Liver transplant, Balloon occlusion, Hypoperfusion, Splenic artery

## Abstract

Splenic steal syndrome (SSS) post liver transplant is a potential cause of graft dysfunction in the setting of peripheral hepatic arterial bed resistance and redirection of blood flow to a dominant splenic artery resulting in reduction of hepatic arterial inflow. We report utilization of balloon occlusion of the proximal splenic artery as an objective measure to confirm the diagnosis of SSS in a patient with orthotopic liver transplant followed by successful treatment with proximal splenic artery embolization using Gelfoam and Amplatzer vascular plug. Written informed consent for the publication of this case report was obtained from the patient.

## Introduction

Splenic steal syndrome (SSS) post liver transplant is a condition that leads to graft hypoperfusion due to blood flow shifting from the hepatic artery to the splenic artery, resulting in elevated liver enzyme as well as graft and biliary tree complications [1]. Vascular problems are often risk factors for ischemia and graft failure. In SSS, additional risk factor contributes to potential graft failure including splenomegaly, spleen-liver ratio, and the size of hepatic and splenic arteries pretransplantation [2]. Although the mechanism of SSS in not fully understood, graft artery stenosis and increased portal perfusion with decreased hepatic arterial blood flow are suspected to contribute to its development [3].

First line for screening SSS is Doppler Ultrasound where parameters such as hepatic arterial index and portal venousvelocity are assessed [2]. The treatment for this syndrome, however, varies with different level of complications associated with each procedure. Some of the procedures performed include splenectomy, narrowing stent, coiled embolization, and banding of the splenic artery [2,3]. This case report intends to highlight the predictive power of celiac angiography with balloon occlusion when it comes to Splenic steal syndrome and its outcome relative to embolization.

## Case report

We present a 54 year-old male patient with a history of hypertension, type 2 diabetes mellitus and decompensated alcoholic cirrhosis (Child Pugh score B9) with recurrent ascites and encephalopathy, who underwent deceased donor orthotopic liver transplant. On post op day 2, he had a hepatic artery revision due to kinked hepatic artery anastomosis and high intrahepatic parenchymal resistance on ultrasound. Follow up transplant ultrasound the next day showed decreased velocities in the hepatic arteries. Patient was also noted to have elevated liver function tests (aspartate aminotransferase 235 units/L [nl: 8-40 units/L], alanine aminotransferase 212 units/L [nl: 7-56 units/L], and total bilirubin 4.6 mg/dL [nl: 0.3-1.2 mg/dL]). Patient underwent angiography due to concerns for hepatic artery stenosis or partial thrombosis.

**Fig. 1:**
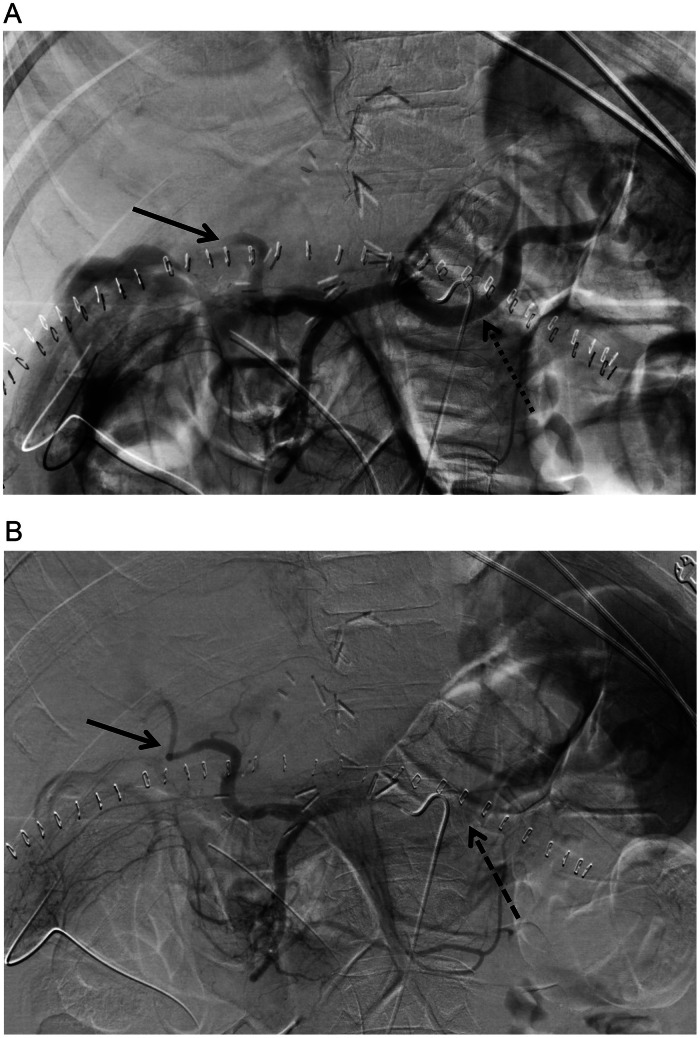
(A) Celiac angiography demonstrates early and brisk filling of the splenic artery (dash arrow) with delayed opacification of the intrahepatic arteries (arrow). (B) Delayed celiac angiography images show persistent slow filling of the intrahepatic arteries (arrow) and early opacification of the splenoportal venous system (dash arrow).

**Fig. 2:**
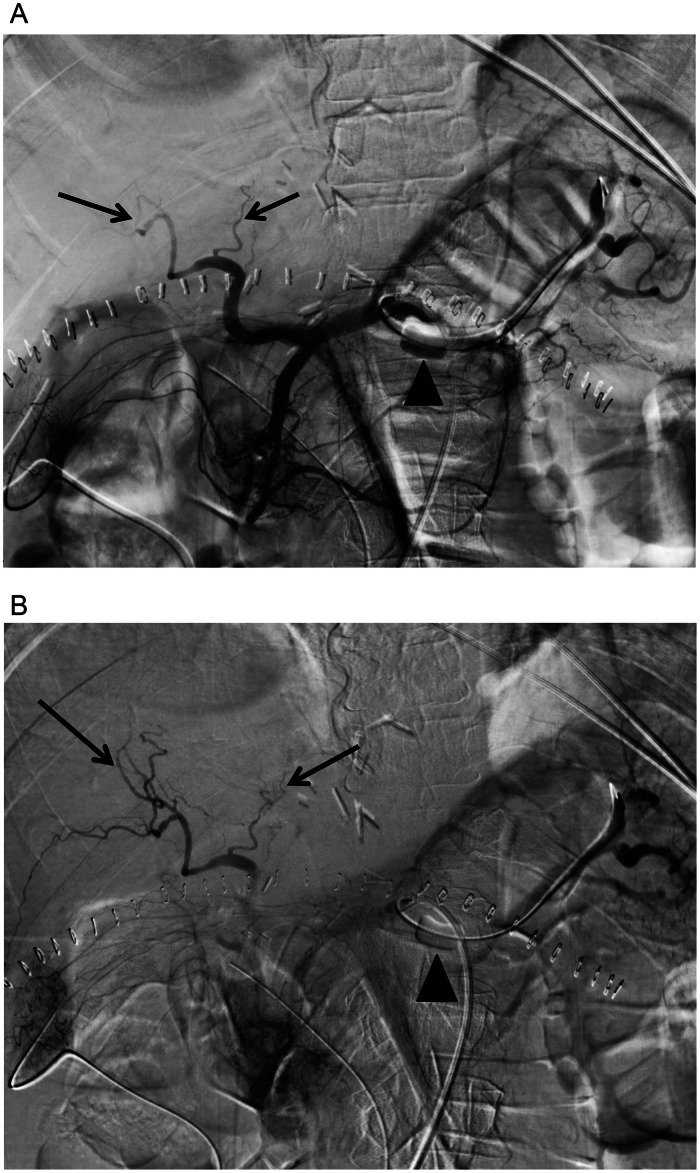
(A) Celiac angiography with balloon occlusion (arrowhead) of the proximal splenic artery demonstrates improved flow and filling of the intrahepatic arteries (arrows). (B) Delayed celiac angiography with balloon occlusion (arrowhead) shows filling of segmental intrahepatic branches (arrows) without early opacification of the splenoportal venous system.

**Fig. 3:**
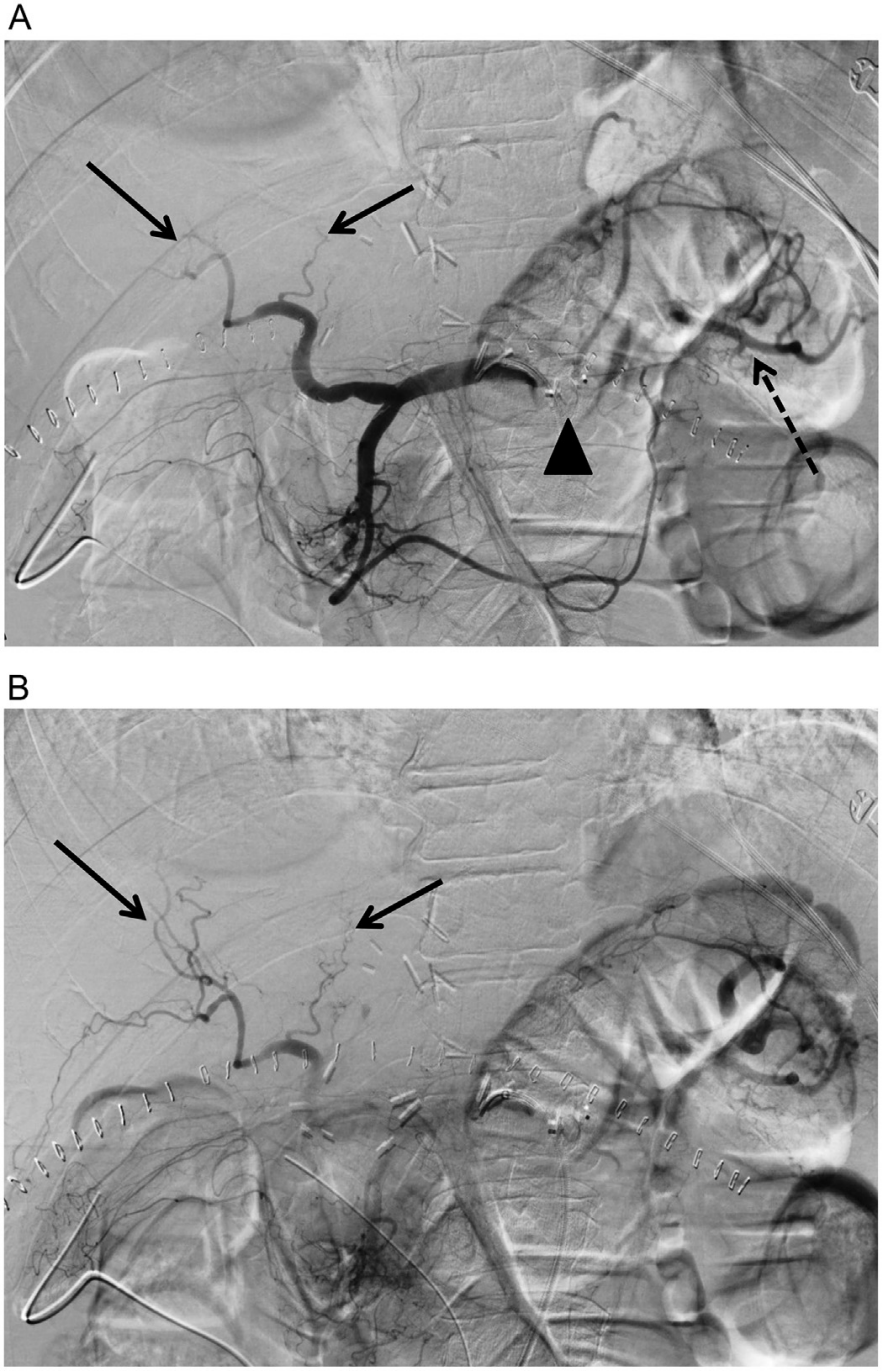
(A) Post embolization celiac angiography shows proximal embolization of the splenic artery using an Amplatzer vascular plug (arrowhead) with satisfactory stasis and delayed filling of the distal splenic artery (dash arrow) via collaterals. There is improved flow and filling of the proximal intrahepatic arteries (arrows). (B) Delayed post embolization celiac angiography shows contrast filling the segmental intrahepatic arteries (arrows) without early opacification of the splenoportal venous system.

Celiac artery angiography using a 5 French VS2 catheter (Cook Medical Inc., Bloomington, IN) showed patent common hepatic artery and proper hepatic artery anastomosis; however, there was decreased caliber and delayed filling of the right and left intrahepatic arteries (Fig. 1). Brisk antegrade flow within the gastroduodenal artery, splenic artery, and gastric artery branches with early filling of the splenoportal venous system noted on angiogram. There was no evidence of hepatic artery stenosis or thrombosis. Given concerns for SSS, the decision was made to perform diagnostic confirmation with balloon occlusion of the splenic artery. A Cook 7 French Flexor Ansel 2 Guiding Sheath (Cook Medical Inc., Bloomington, IN) was inserted into the celiac artery. A 5.5 French Fogarty Thru Lumen embolectomy catheter (Edwards Lifesciences, Irvine, CA) was placed into the proximal splenic artery. The Fogarty Thru Lumen embolectomy catheter was insufflated, and then celiac angiography via the sheath with simultaneous occlusion of the proximal splenic artery (Fig. 2) was performed showing significant improvement in flow and contrast filling of the right and left intrahepatic arteries. After confirming the diagnosis of SSS, the sheath was advanced into the proximal splenic artery and a 10 mm Amplatzer vascular plug II (AVP: St. Jude Medical Inc., St. Paul, MN) was deployed between the origins of the dorsal pancreatic artery and pancreatic magna artery. Additional Gelfoam slurry was injected proximal to the Amplatzer vascular plug II to occlude its interstices. Postembolization celiac angiography demonstrated proximal splenic artery embolization with satisfactory stasis and delayed filling of the distal splenic artery via collaterals (Fig. 3). Post embolization, improved flow was observed with contrast filling the right and left intrahepatic arteries. Repeat transplant ultrasound the following day demonstrated improvement of arterial flow to the liver. Clinically, the liver function tests trended down, and were normalized after 4 days.

## Discussion

Splenic steal syndrome (SSS) is an uncommon complication that affects 0.1% to 10.1% of liver transplant recipients, which can lead to organ ischemia and eventually graft failure [1,4,5]. It is a nonocclusive hypoperfusion of the hepatic arteries due to preferential blood flow to the splenic artery [1,[4], [5], [6], [7]]. While its exact hemodynamic mechanism has not been fully understood, it is speculated to be caused by increased flow to an enlarged splenic artery in the setting of hypersplenism related to prior portal hypertension [4,5,7].

SSS is a diagnosis of exclusion which currently does not have an objective test that is both sensitive and specific [1,4,5,7]. The diagnosis is based on clinical suspicion and imaging findings [1,4,5,7]. Clinical features that suggest SSS and potential graft failure include ascites, biliary necrosis, and elevated liver function tests [1]. Doppler ultrasound is used to screen for SSS, but is unable to differentiate between hepatic arterial strictures and hypoperfusion. These nonspecific findings can show high resistance hepatic artery waveforms, low diastolic flow, diastolic flow reversal, and elevated resistance index (>0.8) [4,5]. High resistance index is not specific to SSS and can occur secondary to graft rejection, edema, outflow impairment or infection.

Celiac angiography is still the gold standard for diagnosis and is commonly performed in order to rule out hepatic artery stenosis [1,4,5]. Angiographic findings supporting SSS include patency of the hepatic artery or lack of anatomical abnormalities, with sluggish or delayed flow relative to the splenic artery [1,5]. However, these findings can be subjective and sometimes inconclusive. Celiac angiography has limited predictive ability at assessing improvement of hepatic arterial flow post splenic artery embolization. As a result, balloon occlusion angiography can be utilized as an objective test of simulating proximal splenic artery embolization and evaluating whether reducing preferential splenic artery flow leads to redirection of flow to the post-transplant hepatic artery [6]. Similar to Saad et al. [6], there was improved flow in the intrahepatic arteries following splenic artery balloon occlusion, however, we performed simultaneous splenic artery embolization in the same session. This prevents the patient from needing a separate treatment session. In this case, the findings after balloon occlusion and splenic artery embolization were similar, suggesting that balloon occlusion is a good objective test to confirm SSS and predict the response to embolization.

## Conclusion

For patients with clinical and ultrasound findings of Splenic steal syndrome post orthotopic liver transplant, performing balloon occlusion of the proximal splenic artery during celiac angiography can help confirm the diagnosis and predict the response to splenic artery embolization.

## Patient consent

Written informed consent for the publication of this case report was obtained from the patient.
